# Practical Notes and Formulæ

**Published:** 1883-08-20

**Authors:** 


### PRACTICAL NOTES AND FORMULÆ.

**Compound Syrup of Wild Cherry.**—F. H. Hazelton (Bridgeton, Maine), in answer to an inquirer, suggests that the preparation may be the following:

| | |
|---|---|
| Fluid extract of wild cherry................ | 2½ fl. ounces, |
| " " " ipecac..................... | ½ " |
| " " " blood-root................ | ½ " |
| Sulphate of morphine...................... | 8 grains, |
| Tartar emetic............................. | 2 " |
| Simp. syrup, sufficient to make............ | 1 pint. |

The same correspondent says that a syrup claimed to be made according to the foregoing formula is extensively sold and prescribed in the State of Maine.—*Ex.*

**Blackberry Cordial.**—

| | |
|---|---|
| Dried blackberries........................ | 16 ounces troy, |
| Or fresh blackberries, ................... | 4 pints, |
| Powdered blackberry root................ | 12 ounces, |
| Powdered mace........................... | 1½ " |
| Powdered cassia.......................... | 9 drachms, |
| Powdered allspice and cloves, of each...... | 5 " |
| Sugar.................................. | 60 ounces troy, |
| Brandy................................. | 2 pints, |
| Port wine............................... | 1½ " |
| Alcohol................................. | 1 " |
| Water.................................. | q. s. |

Soak the berries, if dry, in q. s. of water, and express, and repeat until 6½ pints of juice are obtained. If the berries are fresh, express the juice, and mix water with residue, to wash out all the juice; then add water to make it measure 6½ pints. Mix the spirit with the 6½ pints of juice; moisten the powders with this mixture, and pack in a percolator. Allow it to drain, and pour on water until percolate measures 10 pints; then add the sugar, dissolve, and, if necessary, filter.—*The Pharmacist.*

**New Use of Chloral.**—B. Bonatti recommends the following mixture as an easily administered, prompt and certain drastic purgative:

| | | | | |
|---|---|---|---|---|
| ℞ | Infusi sennæ......... | 300.0 gms. | = | 10 fl. oz. |
| | Chloral............... | 1.5 to 3.0 gms. | | 24 to 45 grs. |
| | Syrupi............... | 30.0 gms. | | 1 fl. oz. |

The infusion of senna is to be prepared from 90 grs. (increasing, if necessary, to 180 grs.) of senna leaves. The author states that he obtained results with this mixture, after jalap and croton oil had failed to act.—*D. Med. Zeit.*

**Hop Cordial.**—The following is recommended as a palatable preparation, not inferior to many of the so-called "Hop Bitters":

| | |
|---|---|
| Hops | 2 ounces, |
| Dandelion | 2 " |
| Gentian | 2 " |
| Chamomile | 2 " |
| Stillingia | 2 " |
| Orange peel | 2 " |
| Alcohol, water, of each | 77 fl. oz., |
| Syrup, simple | 12 fl. oz. |

Exhaust the solids with the alcohol and water, and add the syrup.—*Pop. Science Monthly.*

**Brown's Troches.**—

| | |
|---|---|
| Extract of licorice, powdered | 2 drachms, |
| Sugar, powdered | 3 ounces, |
| Cubebs, powdered, acacia, powdered, each | ½ " |
| Fluid extract of conium | ½ " |

Beat into a thick paste, and cut into lozenges.

(This is said to be the formula, according to *The Pharmacist.*)

**Laxative in Childbed.**—Our women have grown rebellious to the old-fashioned laxative, castor oil, as a purgative for lying in women. It is not now considered necessary or advisable to prescribe a purgative the day following the birth of the child, as was formerly the custom. It were better to wait until the third or fourth day, and then open the bowels with an enema, or give a pill of the following combination which will be found mild and efficient as a laxative, and one which does not affect the milk to the injury of the babe—

| | |
|---|---|
| ℞ Soc. aloes | grs. xv, |
| Ext. nux vomica | grs. v, |
| Pulv. ipecac | grs. iij, |
| Ext. hyosciami | grs. v. |

Make pills No. x.

M. Dose, one pill. If taken at bed-time will act pleasantly next day.

We have used with good effect Warner & Co's Aloin Parvules in such cases. Usually about 4 or 5 of these parvules will be found sufficient to open the bowels in a mild yet efficient manner. The neatness, the small size and the beauty of these little pills make them attractive and acceptable to the most delicate stomach, and the same may be said of the entire list and variety of those beautiful parvules, as prepared by Wm. R. Warner & Co.

**Flatulent Dyspepsia.**—The sulpho-carbolate of sodium in doses of 30 grains, after meals, is recommended as a superior remedy in flatulent dyspepsia. It is also useful in 10 gr. doses for nausea and vomiting, particularly in the vomiting of pregnancy.

**For Pain Preceding the Menstrual Flow.**—For the relief of acute pain in the pelvic region, which not infrequently occurs in women for a day or two antecedent to the occurrence of the menstrual flow, which is, no doubt, due to hyperæmia and hyperæsthesia of the reproductive organs, the Virginia Medical Monthly recommends the following:

℞ Codeiæ sulph.................................gr. j,

Chloral hydrate, } aa.......................grs. xx,

Ammonia bromidi, }

Aquæ camphoræ.............................℥ j.

M. Sig. For one dose, take at bedtime. A repetition of the dose after that time is rarely necessary. In some cases, a warm sitz-bath, for fifteen minutes before retiring, may be taken with advantage.—*Pittsburg Medical Journal.*

**Oxide of Zinc in Chronic Diarrhœa.**—M. Gubler has found it most useful in the diarrhœa of phthisis, and whenever ulceration of the uterus is suspected. He gives it in powders in the form:

℞ Oxide of zinc.............................xxx grs.
Bi-carbonate of soda.......................x grs.

In four powders, two or three daily.—*Summary.*

**Liniments in Muscular Rheumatism.**—The following preparations are recommended in a recent issue of the Gazette Medicale de Paris:

℞ Saponis....................................ʒ iss.

Ætheris acetic..............................℥ j.

Camphoræ...................................ʒ j.

M.

Or,

℞ Tr. aconit. rad..............................m. l.

Ung. simplicis..............................℥ j.

Misce bene et adde.

℞ Chloroform.................................ʒ iss.
Morphiæ hydrochlorate.......................gr. 1-5
M.—*Med. and Surg. Rep.*

**Dysmenorrhœa Mixture.**—

Iodide of potassium...................... 2 drachms.
Sulphate of iron......................... 1½ "
Distilled water.......................... 2 ounces.
Tr. of cardamom.......................... 4 drachms.
Syrup of ginger..........................12 "

Mix; dose, a teaspoonful three times a day, in a little water. Use two weeks before the period, for two periods.—*Medical and Surgical Reporter.*

